# Using Formative Research to Design an Epidemiologic Survey: The North Carolina Study of Home Care and Hospice Nurses

**DOI:** 10.4178/epih/e2011008

**Published:** 2011-09-07

**Authors:** Jack K. Leiss, Jennifer T. Lyden, Cynthia Klein

**Affiliations:** Constella Group, Durham, NC, USA.

**Keywords:** Data collection, Focus groups, Nurses, Questionnaires, Surveys

## Abstract

**OBJECTIVES:**

Formative research can serve as a means of obtaining important information for designing an epidemiologic study, but descriptions of this approach in the epidemiologic literature are lacking. The objective of this paper is to describe the use of three formative research techniques in designing a survey of home care and hospice nurses.

**METHODS:**

We conducted two focus groups, seven key informant interviews, and approximately fifteen hours of direct observation among home care and hospice nurses recruited by word of mouth in North Carolina in 2006.

**RESULTS:**

We used information obtained from the formative research to decide which survey design would likely be most successful with this population (mail survey, as opposed to Internet survey or in-person interviews), which measure to use for the denominator of the blood exposure incidence rates (number of visits, as opposed to patient-time), and which items and response options to include in the questionnaire, as well as to identify specific survey techniques that would likely increase the response rate (emphasizing the regional focus of the study; sending the questionnaire to the home address).

**CONCLUSION:**

When particular information for planning a study is unavailable from the literature or the investigator's experience, formative research can be an effective means of obtaining that information.

## INTRODUCTION

Nurses who provide care in the home are at risk of infection with human immunodeficiency virus (HIV), hepatitis B virus (HBV), and hepatitis C virus (HCV) from blood exposure brought about by needlestick, blood contact with the mucous membranes of the eyes, nose, and mouth, or blood contact with non-intact skin. This issue is important because the population is large (over 130,000 nurses worked in home care/hospice in the United States in 2009) and expected to grow as home care/hospice expands [1]. In order to gain information for preventing blood exposure in this population, a survey was conducted among home care/hospice nurses in the state of North Carolina. This paper describes the formative research that was conducted as part of the planning phase for that survey.

Epidemiologists generally rely on familiarity with the study population, based on the investigator's experience and guidance from the literature, to select the best recruitment and data collection methods for a particular study. However, in the absence of sufficient prior knowledge from these sources, formative research can provide information on how a particular population is likely to respond to alternative methods of recruitment and data collection [2]. Additionally, formative research can be used to inform the design of the data collection instruments [3-8].

Formative research methods are commonly applied in other research disciplines [9-13], but they are rarely included in epidemiologic study reports [14]. Although their use by epidemiologists may be greater than these reports indicate, descriptions of how to use formative research for the above purposes are lacking in the epidemiologic literature [15,16]. The purpose of this paper is to describe the use of three formative research techniques, i.e., focus groups, key informant interviews, and direct observation, in designing the survey of home care and hospice nurses.

The North Carolina Study of Home Care and Hospice Nurses, conducted in 2006, was a mail survey of factors related to occupational blood exposure in this population [17]. The specific aims of the study were to estimate incidence rates of blood exposure, identify risk factors for blood exposure, and quantify the availability and use of personal protective equipment and medical safety devices (provision of which is intended to prevent blood exposure). The only comparable previous population survey of non-hospital health care workers was a national study of paramedics conducted by our team [18]. Our preliminary investigation for the nurses study suggested that factors related to blood exposure, as suggested by the types of medical care provided and the characteristics of the work environment [19,20], would be different for the nurses compared to paramedics, although there would be some overlap. Similarly, differences between the two study populations-paramedics vs. registered nurses, national vs. state target populations, younger males with less professional experience (paramedics) vs. older females with more experience (nurses), considerable unstructured time at work between calls (i.e., for paramedics to respond to the survey) vs. little or no free time during work hours (nurses)-suggested that different factors would influence participation in the study of nurses compared to the earlier study of paramedics.

Given the need to better understand the above aspects of the study population before finalizing the study design and the unavailability of this information from other sources, we conducted formative research to guide the planning of the survey. The objectives of the formative research were to 1) inform the development of selected questionnaire items and response options, 2) determine the best method of data collection (mail, telephone, or Internet-based survey or in-person interviews), and 3) identify barriers, incentives, and motivations that would affect participation in the study. The application of these techniques and what was learned are described below.

## MATERIALS AND METHODS

Home care and hospice nurses from the four types of home care or hospice agencies (i.e., hospital-based, private freestanding, health department, and hospice) that operate in North Carolina, a largely rural state in the southeastern United States, were recruited to participate in the formative research. Because a statistically representative sample is not needed for formative research, convenience samples were used. One of the investigators (Lyden JT) called the home nursing coordinators of various agencies, who were unfamiliar with the study and the study team, and explained the purpose of the focus group, key informant interview, or direct observation (depending on which one was being recruited for) and the type of participants needed. The home nursing coordinator then recruited the participants. No structured selection process was followed in choosing the agencies to contact; however, different types of agencies were contacted to ensure participation from home care and hospice nurses from the four types of agencies listed above. For example, one agency was contacted through a friend who knew the nursing coordinator.

Because the survey would be restricted to nurses who were registered nurses (RNs), the formative research was likewise restricted to RNs. In addition, the formative research participants were all white middle-aged females with several years' experience in home nursing (although only the experience was specified to the nursing coordinators as a selection criterion). This reflects the characteristics of the survey target population, which was predominantly white (89%) females (95%) aged 36-55 years (62%) [17]. All participants were given a $25 (key informant interview and direct observation) or $50 (focus group) gift card. Participants were assured that their participation was voluntary and confidential. This study was approved by the Institutional Review Board (IRB) of Weber State University.

### Focus groups

We conducted two focus groups [21], one in a rural area and one in an urban area, to obtain information on the feasibility of alternative data collection methods (Objective 2) and barriers and incentives to participation in the study (Objective 3). One focus group had five participants and the other had nine participants. All participants were nurses who conducted home visits, as opposed to supervisors, shift managers, or administrators. In accordance with human subjects protection criteria, signed informed consent was not obtained because it would have constituted the only record of the participants' identity.

One of the authors (Lyden JT), a trained focus group moderator, facilitated all focus group discussions while a co-moderator took notes. Each session lasted approximately 60 minutes. The discussions were audio recorded.

### Key informant interviews

We conducted key informant interviews [21] with seven nurses to inform development of questionnaire items and response options (Objective 1). All of the participants had at least 10 years experience in home care/hospice. At the time of the interviews, some of the participants were mangers of nurses who made home visits. At least one key informant was from each of the four types of home care/hospice agencies listed above. All interviews were conducted by one of the authors (Lyden JT). Interviews lasted 60 to 90 minutes each. The nurses who participated in the key informant interviews did not participate in the focus groups. The questions addressed in the key informant interviews are given in Table 1.

### Direct observation

We conducted direct observation (also called shadowing) [22] to inform development of questionnaire items and response options (Objective 1). Three nurses, one each from a hospice agency, a county health department, and a private freestanding agency, participated. One of the authors (Lyden JT) accompanied the nurses on their respective shifts (approximately 4-6 hours each) to directly observe their work environment and activities. Eight home visits were observed. The visits were conducted in rural, urban, and suburban homes and lasted 20 to 55 minutes each.

## RESULTS

### Objective 1: Questionnaire items and response options

We used information obtained from key informant interviews and direct observation to formulate questions and response options for the survey instrument. The specific issues that were addressed by the formative research were 1) which routes of blood exposure are relevant for this population; 2) which measures for the denominator of the incidence rates are feasible to obtain in this study; 3) which skilled medical procedures are routinely performed by this population; and 4) which types of personal protective equipment and safety-engineered medical devices are relevant for this population.

As shown in Table 1, the formative research found that needlestick, blood on non-intact skin, and blood in eyes, nose, or mouth are the three routes of blood exposure that are relevant for this population and that number of visits in a typical week, averaged over different seasons, was the denominator measure that was feasible to obtain for the survey. In addition, the skilled medical procedures that are routinely performed in home care/hospice nursing and the types of personal protective equipment and safety-engineered medical devices that are relevant for this population were identified. The questionnaire that was developed from this information and subsequently used in the survey can be viewed at http://www.sra.com/nchhnquestionnaire/.

### Objective 2: Determine the best method of data collection for the home care and hospice nurse population

Four different methods of data collection were presented to the focus groups for their reactions.

#### Telephone survey

All of the focus group participants thought that a telephone survey would be unacceptable because it would have to be completed during non-work hours. Many participants indicated that they did not want to be disturbed in the evening by a telephone survey. Nurses consistently mentioned that they were very busy and had little or no "free-time." When asked their initial thoughts about a telephone survey, responses included, "I would hang up", "I don't answer calls from numbers that I don't recognize", "No, no, not a phone survey", and "There is never a good time to reach us."

#### In-person interviews

Focus group participants unanimously agreed that in-person interviews were impractical for this survey. Nurses would not feel safe being interviewed in their homes. Similarly, they would not want to be interviewed at their workplaces out of concern that their employers would hear their blood exposure information. Home care/hospice nurses are required to report occupational blood exposures to their employers, but they do not always do so. They did not want their employers learning of unreported exposures through interviews conduced at the workplace.

#### Internet survey

Many of the participants did not have Internet access or were not interested in completing a questionnaire online. Many participants did not know how to use the Internet or computers. These responses perhaps reflect the lack of Internet access in some rural areas and the age of the participants, many of whom may have been practicing in their profession for some years before computerized work practices were introduced. Participants were also concerned about the security of data transmitted over the Internet.

#### Mail survey

Focus group participants consistently agreed that a mail survey would be the best method of data collection. Participants indicated that a mail survey would allow the nurses to complete the questionnaire at a time of their choosing. Furthermore, nurses suggested mailing the survey to the home addresses. One nurse said, "I don't check my mailbox at work." Additionally, mailing surveys to the home addresses would alleviate their concerns about their employers learning of unreported blood exposures.

### Objective 3: Barriers, incentives, and motivations for participating in the study

#### Barriers

Focus group participants consistently mentioned two barriers to participation, lack of time and concern about the confidentiality of their exposure data. To address these barriers, participants suggested using a mail survey sent to the home address. Nurses were not concerned about the research team knowing their exposure status. However, the nurses told us explicitly not to ask the name of their employer.

#### Incentives

Nurses suggested that the inclusion of an incentive would increase participation in the study. All nurses agreed that an incentive should be included with the questionnaire. The most frequently mentioned incentive was cash; however, non-cash incentives were also suggested. The monetary value of the suggested incentive ranged from a dollar to thousands of dollars (for example, a luxury item such as a vacation trip or a vehicle, that would be awarded to one of the survey participants by lottery).

#### Motivations

Participants identified numerous factors that would motivate nurses to participate in the study, including empowerment, the potential for drawing attention to the issue of occupational blood exposure, and the hope that the results would reduce future exposures. In addition, the study would bring recognition to North Carolina home care and hospice nurses. Many nurses indicated that the regional focus of the study was appealing. One nurse said, "It is great that information will be gathered on our state." The nurses noted that the appearance of the mail survey materials (outgoing envelope, cover letter, questionnaire layout and cover, and return envelope) would affect the perceived credibility of the study. The mail survey packet should not appear to be direct marketing materials.

Participants suggested that the survey be easy to read, include space to write comments, easy to follow, and be no more than two pages in length. Participants identified the purpose of the study, length of time to complete the survey, topic being studied, and number of nurse participants as issues that they would use to determine participation.

Table 2 presents selected questions from the focus group moderator's guide that addressed Objective 3.

## DISCUSSION

For researchers who are unfamiliar with their study population, formative research provides a means of gaining information with which to design the recruitment and data collection phases of the study. We described the use of this approach and its contribution to the design a survey of home care and hospice nurses.

### Major study design issues

The question of which data collection method will be most effective in achieving study objectives depends, to a large extent, on characteristics that are particular to the specific population being studied. We did not find any published studies of home care/hospice nurses that suggested how our population would respond to any of the different methods of data collection available to us. Our a priori preference was to conduct either an Internet or interview (in-person) survey. If we had proceeded with that plan, we would have learned in the pilot study that it was not feasible. By obtaining this crucial information in the formative research phase, we avoided the substantially greater cost and delay of having to redesign the survey following the pilot study. Furthermore, after such an ill-fated pilot study, we still would not have known which study design might work for this population. However, based on the information gained in the formative research, we implemented the study as a mail survey [17].

Secondly, we needed to determine the most appropriate denominator for calculating blood exposure incidence rates. This depended in part on the information that the study population would be able and willing to provide. In the previous survey of paramedics, we used number of patients and number of calls (analogous to number of visits for home care/hospice nurses) as denominators for the blood exposure incidence rates [18]. However, because of the widely varying duration of home visits, we initially thought that patient-time would be a more appropriate denominator for incidence rates among home care and hospice nurses. From the formative research, we learned that we could not obtain information on the amount of time spent with each patient, but we could obtain the number of visits in a fixed time period. We also learned that, contrary to the paramedic survey, it was unnecessary to ask about the number of patients seen at each visit because only one patient is seen per visit. Based on this information, we selected number of visits as the denominator [17].

### Questionnaire items

The objectives of the survey required asking several questions for each of the relevant routes of occupational blood exposure. There are five possible routes [18]. However, through the formative research we learned that only three of these routes (i.e., needlesticks, blood on non-intact skin, and blood in eyes, nose, and mouth) are relevant in home care/hospice. This allowed us to have a shorter and more pertinent survey instrument.

Similarly, through the formative research we learned which skilled nursing procedures, types of personal protective equipment, and types of safety devices to include as response options in the questionnaire.

### Maximizing response rate

Various techniques are available for increasing survey response rates, but the ones that will be most effective for a particular study are, in part, specific to the population being recruited [23]. For example, the factors identified by the nurses as likely to motivate increased participation in the study are consistent with the general survey research literature [24]. However, the knowledge that, for this particular population, the regional (North Carolina) nature of the study was a salient feature that could be used to increase participation [25] was available to us only through the formative research. As a result, we included "North Carolina" in the study name, which appeared on the return address of the survey envelope. We also designed a study logo that incorporated the distinctive outline of the state. This logo appeared on the envelope, cover letter, questionnaire, and incentive (a magnet).

### Departures from the formative research findings

There were two suggestions from the formative research that we did not follow. Nurses indicated that the questionnaire should be limited to two pages to achieve a reasonable response rate. We could not meet the study objectives with a two-page questionnaire. The final questionnaire was nine pages.

Secondly, the nurses advised against asking the name of the agency at which the respondent worked. However, we considered this important information. We included that question in the instrument, and it was subsequently answered by 98 percent of respondents. We do not know whether inclusion of that item reduced participation in the survey. The overall adjusted response rate was 69 percent [17].

Formative research can be an effective tool for designing population studies. It can provide basic information about the study population that is lacking from the literature and the investigator's experience. In our study of occupational blood exposure among home care and hospice nurses, we used formative research to identify the routes of blood exposure, medical procedures, and types of personal protective equipment and safety medical devices that were relevant for this population. This information is likely generalizable to other populations of home care and hospice nurses.

Even when the investigator is familiar with the population in general, formative research can provide information on critical factors that are specific to the particular population being studied. For these factors, the results of our study may not be generalizable to other populations. We learned from the formative research that highlighting the regional focus of the study would likely increase participation. In addition, we learned that the study design preferred by the investigators, an Internet or interview survey, would have had low participation. If we had not learned that until the pilot study, time and resources that were needed for implementing the survey would have gone instead to redesigning it, severely impinging upon the success of the project. Overall, the information gained from the formative research was a critical factor in the success of the study.

## Figures and Tables

**Table 1:**
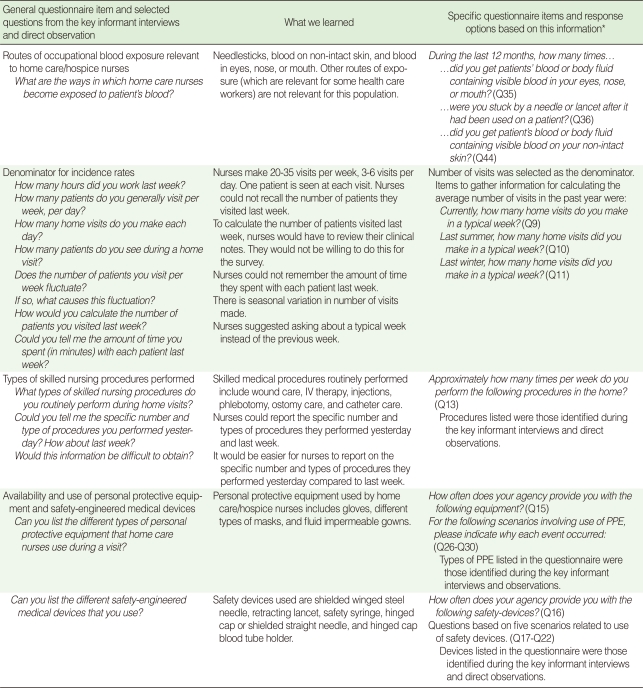
Information gained from key informant interviews and direct observation for designing the questionnaire, North Carolina Study of Home Care and Hospice Nurses, 2006

^*^The questionnaire item number is given in parentheses.

**Table 2:**
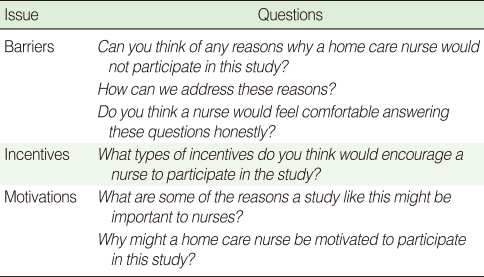
Selected questions from the focus group moderator's guide regarding barriers, incentives, and motivations for participating in the survey, North Carolina Study of Home Care and Hospice Nurses, 2006

## References

[1] Bureau of Labor Statistics May 2008 national industry-specific occupational employment and wage estimates. NAICS 621600: home health care services. Occupational employment statistics.

[2] Costenbader KH, Brome D, Blanch D, Gall V, Karlson E, Liang MH (2007). Factors determining participation in prevention trials among systemic lupus erythematosus patients: a qualitative study. Arthritis Rheum.

[3] Moyer CA, Arnold L, Quaintance J, Braddock C, Spickard A, Wilson D (2010). What factors create a humanistic doctor? A nationwide survey of fourth-year medical students. Acad Med.

[4] Byrd TL, Chavez R, Wilson KM (2007). Barriers and facilitators of cervical cancer screening among Hispanic women. Ethn Dis.

[5] Reece M, Milhausen RR, Perera B (2006). A theory-based approach to understanding sexual behavior at Mardi Gras. J Sex Res.

[6] Kim JR, Fisher MJ, Elliott D (2006). Attitudes of intensive care nurses towards brain death and organ transplantation: instrument development and testing. J Adv Nurs.

[7] Tam Ashing K, Padilla G, Tejero J, Kagawa-Singer M (2003). Understanding the breast cancer experience of Asian American women. Psychooncology.

[8] Wilson TD, Brenner M, Brown J, Canter D (1985). Questionnaire design in the context of information research. The research interview: uses and approaches.

[9] Flores AL, Prue CE, Panissidi P, Lira A (2010). Preparing for a healthy future today: Folic acid formative research with young Latina adults. Fam Community Health.

[10] Morrison RS, Peoples L (1999). Using focus group methodology in nursing. J Contin Educ Nurs.

[11] Gong F, Baron S, Ayala L (2009). Formative research in occupational health and safety intervention for diverse, underserved worker populations: a homecare worker intervention project. Public Health Rep.

[12] Sigström R, Skoog I, Sacuiu S, Karlsson B, Klenfeldt IF, Waern M (2009). The prevalence of psychotic symptoms and paranoid ideation in non-demented population samples aged 70-82 years. Int J Geriatr Psychiatry.

[13] Gittelsohn J, Evans M, Helitzer D, Anliker J, Story M, Metcalfe L (1998). Formative research in a school-based obesity prevention program for Native American school children (Pathways). Health Educ Res.

[14] Linet MS, Byrne J, Willis GB, Wacholder S, Forman MR (2007). Maternal sensitivity concerning aetiological research into childhood cancer: results of preliminary focus groups. Paediatr Perinat Epidemiol.

[15] Ford ME, Hill DD, Blount A, Morrison J, Worsham M, Havstad SL (2002). Modifying a breast cancer risk factor survey for African American women. Oncol Nurs Forum.

[16] Nichter M, Nichter M, Thompson PJ, Shiffman S, Moscicki AB (2002). Using qualitative research to inform survey development on nicotine dependence among adolescents. Drug Alcohol Depend.

[17] Leiss JK, Lyden JT, Mathews R, Sitzman KL, Vanderpuije A, Mav D (2009). Blood exposure incidence rates from the North Carolina study of home care and hospice nurses. Am J Ind Med.

[18] Leiss JK, Ratcliffe JM, Lyden JT, Sousa S, Orelien JG, Boal WL (2006). Blood exposure among paramedics: incidence rates from the national study to prevent blood exposure in paramedics. Ann Epidemiol.

[19] Leiss JK, Sousa S, Boal WL (2009). Circumstances surrounding occupational blood exposure events in the National Study to Prevent Blood Exposure in Paramedics. Ind Health.

[20] Sitzman KL, Leiss JK (2009). Documentation of incidental factors affecting the home healthcare work environment. Home Healthc Nurse.

[21] Patton MQ (1990). Qualitative evaluation and research methods.

[22] Duray CG, Wilson JR, Corlett EN (1990). Methods for direct observation of performance. Evaluation of human work.

[23] Groves RM, Fowler FJ, Couper MP, Lepkowski JM, Singer E, Tourangeau R (2009). Survey methodology.

[24] Edwards P, Roberts I, Clarke M, DiGuiseppi C, Pratap S, Wentz R (2002). Increasing response rates to postal questionnaires: systematic review. BMJ.

[25] Groves RM, Singer E, Corning A (2000). Leverage-saliency theory of survey participation: description and an illustration. Public Opin Q.

